# Congenital Vertical Talus: An Updated Review

**DOI:** 10.7759/cureus.45867

**Published:** 2023-09-24

**Authors:** Jonathan Day, Ryan S Murray, Sarah Dance, Correggio L Peagler, Sean Tabaie

**Affiliations:** 1 Orthopaedic Surgery, Georgetown University Medical Center, Washington DC, USA; 2 Orthopaedic Surgery, Children’s National Hospital, Washington DC, USA; 3 Orthopaedic Surgery, The George Washington School of Medicine and Health Sciences, Washington DC, USA; 4 Orthopaedic Surgery, Children's National Hospital, Washington DC, USA

**Keywords:** congenital convex pes valgus, congenital valgus flatfoot, orthopaedic surgery, rocker-bottom flatfoot, congenital vertical talus

## Abstract

Congenital vertical talus (CVT) is the presence of rigid flatfoot deformity characterized by hindfoot valgus and equinus. This foot deformity is associated with midfoot dorsiflexion and forefoot abduction due to a fixed dorsal dislocation of the navicular relative to the head of the talus. It is often underdiagnosed in children due to its similarity to other disorders of the foot. Misdiagnosis of CVT and subsequent failure to address it leads to significant disability and pain. While past surgical management consisted of soft tissue releases that produced varying efficacy, current management of CVT consists of serial casting and minimally invasive procedures that have yielded excellent long-term outcomes. This review provides insight into the diagnosis and treatment of CVT with the intention of highlighting the importance of promptness of intervention to prevent further disability.

## Introduction and background

Congenital vertical talus (CVT) is a rare congenital foot deformity, characterized by its rigidity and co-occurrence with other neuromuscular disorders. Often referred to as “rocker-bottom flatfoot”, CVT is distinguished by a persistent dorsal dislocation of the talonavicular joint at the head of the talus. In turn, this results in hindfoot equinovalgus, midfoot dorsiflexion, and forefoot abduction [1]. A challenging aspect of CVT is its propensity to be mistaken for other prevalent, more benign positional foot deformities, resulting in frequent misdiagnoses or missed cases [1,2]. This consequently raises the risk of disabling sequelae, such as severe pain and medial plantar callus formation, which is associated with talar head prominence [1,3,4].

The primary objective of surgical intervention for CVT is the restoration of the anatomical alignment of the foot and ankle, facilitating physiologic movement and function. Historically, surgical management predominantly involved the use of extensive soft tissue releases. However, these resulted in prolonged postoperative recovery periods from complications, including over- and under-correction of the original deformity [5]. Furthermore, the scar tissue from these releases often leads to pain and stiffness over time. Recent advancements in our understanding of the pathophysiology of CVT have paved the way for more effective treatment strategies that involve serial casting and minimally invasive surgical procedures, yielding outstanding long-term outcomes for these patients [1-5].

CVT was first described by Rocher in 1913 [6] and has since been referred to by many terms including reversed club foot, congenital valgus flat foot, and rocker bottom foot. In modern literature, it is most commonly referred to as congenital convex pes valgus [7]. The estimated prevalence of this disorder is 1 in 10,000 live births [1]. This condition exhibits no discernible gender preference and has unknown incidence [8], likely due to frequent missed diagnoses in neonates. In most cases, the etiology of CVT remains elusive, although roughly half of all cases occur concurrently with neuromuscular disorders or syndromes. Among these, the most prevalent are arthrogryposis, myelomeningocele, and Marfan’s Syndrome [1,9,10]. Additionally, less common neuromuscular associations include cerebral palsy, spinal muscular atrophy, and polio. CVT also has notable associations with aneuploidy of chromosomes 13, 15, and 18 [1,11]. Despite nearly 50% of all CVT cases being classified as idiopathic, approximately 20% of these cases have a positive family history, which may be attributed to their autosomal dominant inheritance with incomplete penetrance [1,12].

**Figure 1:**
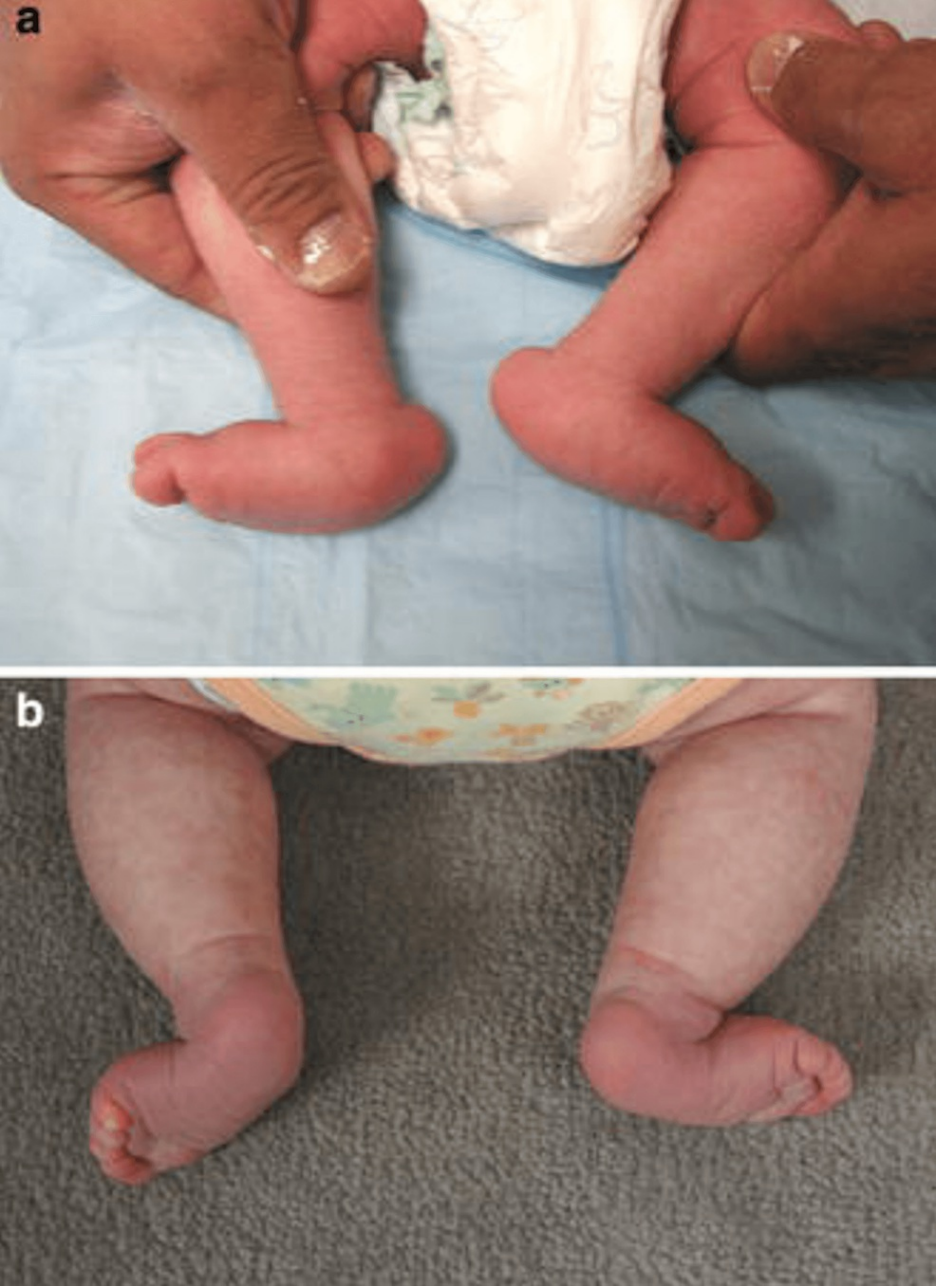
Clinical photographs demonstrating the features of congenital vertical talus. a) Bilateral congenital vertical talus deformities in a six-week-old infant demonstrating the convex plantar surface of the feet. b) Deep creases are present on the dorsolateral aspect of the foot in an eight-week-old infant with bilateral congenital vertical talus Figures courtesy Alaee et al. [13]. Creative Commons License (CC BY 4.0).

The association between CVT and other neuromuscular disorders can be explained by muscle imbalances that promote abnormal forces on the foot, leading to deformity. The particular imbalances seen are contingent on the patient’s underlying neuromuscular disorder. For example, in patients with myelomeningocele with associated CVT, the deformity is contributed by relatively strong ankle dorsiflexors acting against weak posterior tibialis [1]. Other neuromuscular disorders may cause intrinsic muscle weaknesses that lead to CVT. Conditions related to central nervous system dysfunction tend to manifest more rigidity given they are secondary to profound muscle imbalances. This imbalance is a hallmark in congenital foot and ankle deformities (Figures 1A, 1B), and often culminates in enduring sequelae if not promptly addressed.

## Review

Pathoanatomy

At the core of the pathophysiology of many complex foot and ankle disorders lies the delicate interplay among the many joints and soft tissue structures. A hallmark characteristic of CVT is its hindfoot equinovalgus, stemming from a combination of Achilles tendon contracture and constriction of the posterolateral ankle and subtalar joint capsules. Moreover, contractures of several muscles, including tibialis anterior, extensors digitorum and hallucis longus, extensor hallucis brevis, peroneus tertius, and the dorsal talonavicular capsule, induce dorsiflexion and abduction of the midfoot and forefoot respectively to the hindfoot [1].

Characteristic osseous abnormalities include a dorsal and lateral dislocation of the navicular relative to the talar head, creating a hypoplastic, wedge-shaped navicular [1]. Dorsal flattening of the talar head and neck are adaptations due to displacement of the navicular head [14]. In addition, the talus becomes vertically oriented with a prominent talar head oriented inferiorly. This prominence leads to impingement and attenuation of the ligaments situated on the plantar surface of the talocalcaneonavicular joint. This process causes stretching and weakening of the calcaneonavicular (spring) ligament and anterior fibers of the deltoid ligament, giving rise to the distinctive rocker-bottom appearance [14]. The plantar aspect of the foot appears convex, while the dorsal aspect of the midfoot develops a profound crease [1]. As a consequence of the vertical talus, the calcaneus is forced into plantarflexion, often leading to either dorsolateral subluxation or complete dorsal dislocation of the calcaneocuboid joint. Furthermore, the superior peroneal retinaculum becomes attenuated, resulting in anterior subluxation of the peroneal tendons over the distal fibula. Medially, the posterior tibial tendon also experiences anterior subluxation in proximity to the medial malleolus, becoming attenuated as it traverses onto the plantar surface of the midfoot [14]. Biomechanically, this alters the force vector as the subluxated tendons now function predominantly as ankle dorsiflexors rather than ankle plantar flexors [13,14].

Physical exam

Newborns presenting with a flatfoot deformity require meticulous examination to differentiate CVT from other differential diagnoses, including positional calcaneovalgus deformity, posteromedial bowing of the tibia, congenital absence of the fibula, oblique talus, and idiopathic flatfoot deformity [15]. Newborns with CVT classically present with hindfoot equinovalgus, forefoot abduction, and midfoot dorsiflexion. The rigidity of this rocker-bottom foot deformity is key to distinguishing vertical talus from the aforementioned conditions. The presence of hindfoot equinus is perhaps the most sensitive finding, as the absence of this deformity more likely points to a positional deformity. 

Because of the diverse associations tied to CVT, a comprehensive, head-to-toe physical exam is imperative. The identification of dysmorphic facial features may warrant referral to a geneticist, while observations such as a sacral dimple might prompt MRI evaluation and referral to a pediatric neuromuscular specialist for comprehensive assessment of potential central nervous system anomalies. 

On appearance, the clinician can easily appreciate the characteristic rocker-bottom appearance. Deep dorsal creasing of the foot results in a gap where the navicular and talar head would normally articulate dorsally in a typical foot. Reduction of the gap through forefoot plantarflexion is associated with a more favorable prognosis and improved responsiveness to treatment [1]. The examination also discloses a palpable talus just medial to the sole of the foot. 

In this patient population, serial stimulation of the nerves on the plantar and dorsal aspects of the foot allows for evaluation of motor function in plantarflexion and dorsiflexion, which is important given the central nervous system involvement that is associated with a neuromuscular etiology of CVT [1]. The presence of dorsiflexion and plantarflexion of the toes is recorded as absent, slight, or definitive. This should be recorded for the hallux individually, and the lesser digits collectively. Reduced or absent movement with stimulation correlates to rigid CVT, which is generally more difficult to treat and correlates with a poorer prognosis. Finally, hindfoot equinus contracture can be assessed using a Silfverskiöld-like test. A positive contracture deformity is defined as the inability to achieve 10 degrees of passive ankle dorsiflexion with the knee extended and flexed [1].

Weightbearing can exacerbate a rigid vertical talus deformity by creating adaptive changes in the tarsal bones if left untreated. While the rigid flatfoot deformity of CVT does not delay walking, patients may develop a leg-leg gait in which there is absence of heel strike and limited forefoot push-off power. In fact, the surface area of the foot that is in contact with the ground during weight-bearing is reduced to the size of a half-dollar [15]. In addition, painful calluses can develop along the plantar medial edge of the foot adjacent to the dislocated talar head [1,3].

Radiographic Evaluation

Standard radiographic evaluation of CVT begins with plain foot films in anteroposterior and three lateral views with the foot in maximal dorsiflexion, maximal plantarflexion, and neutral [1]. The lateral view obtained during maximal plantarflexion is most informative in determining the rigidity of the talonavicular dislocation. Older pediatric patients may undergo radiographic standing views.

The assessment of the newborn foot poses distinct challenges. Firstly, their cartilaginous bones make differentiation of CVT from other foot deformities difficult. Secondly, at birth, ossification of the cuneiforms, navicular, and cuboid is absent. Only the hindfoot and metatarsals are visible radiographically at birth [1]. Ossification of the cuneiforms occurs between three months and 2.5 years, with the lateral cuneiform being the first to undergo ossification [14]. The cuboid begins ossifying between six and seven months, while the navicular can take anywhere from nine months to five years to ossify. For this reason, radiographic evaluation of suspected CVT in a newborn must focus on the relationships between the ossified structures. 

**Figure 2:**
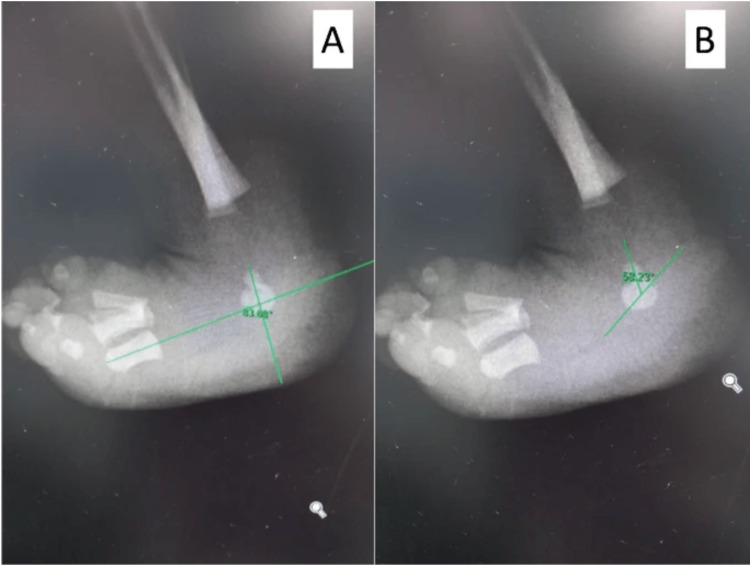
Xray outlining the angles measured A. talo-first metatarsal angle  B. talocalcaneal angle Images courtesy Utrilla-Rodríguez et al. [17] Creative Commons License (CC BY 4.0).

Several crucial radiographic parameters help in assessing CVT. Specifically, on the standard AP view of the foot, the talocalcaneal angle (kite angle), normally falls within the range of 20 to 40 degrees in children less than five years of age [16]. In contrast, patients with CVT typically exhibit elevated tibiocalcaneal angles, indicative of increased hindfoot deformity. Additionally, the talo-first metatarsal angle (Meary’s angle) on the AP view is often greater than 30 degrees, further signifying hindfoot valgus. On the lateral view, critical radiographic parameters include the tibiocalcaneal angle, kite angle, and Meary’s angle (Figure 2A, 2B) [17]. In cases of CVT, the long axis of the talus aligns vertically relative to the first metatarsal, and the calcaneus is affected by severe equinus, thereby increasing the lateral Meary’s, kite, and tibiocalcaneal angles [1].

Dynamic lateral views of the foot in forced, maximal plantarflexion play an important role in assessing rigidity and ruling out other differential diagnoses, such as oblique talus. In particular, the lateral view of the foot in maximal plantarflexion demonstrates persistent vertical orientation of the talus, while in oblique talus, the dorsally subluxated talonavicular joint reduces in plantarflexion [1,18]. Furthermore, maximal dorsiflexion lateral views reveal persistent, rigid hindfoot equinus. An increased talocalcaneal angle is consistently seen in all CVT patients; however, there is not a specific angle measurement that is a pathognomonic indicator for this deformity.

Classification

The existing classification systems for CVT are based on the presence or absence of anatomic abnormalities and any coexisting diagnoses. Among these, Coleman et al. [4] proposed an anatomic classification system that is most widely utilized, dividing CVT into two distinct types. A type 1 deformity is characterized by a rigid dorsal dislocation of the talonavicular joint, while a Type 2 deformity has an additional concomitant dislocation of the calcaneocuboid joint [1,4]. While other classification systems have been proposed in the literature [18], none incorporate lower limb motor function. Reduced or absent motor function of the lower leg and ankle is predictive of poor prognosis, resulting in a higher risk of relapse [9,19]. This suggests that current classification systems for CVT have limited prognostic utility, warranting the need for a revised system that incorporates functional deficits. 

Treatment

The primary objectives of treatment include restoring normal anatomic joint alignment and properly restoring weight bearing on the foot tripod. Since CVT was first described in 1913, the treatment approach has evolved from exclusively nonoperative management to a combination of nonoperative and surgical techniques [14]. In general, surgical intervention is deferred until the child is at least 12 months of age. The current standard of practice involves a minimally invasive approach comprising of manual manipulation of the deformed foot, followed by serial casting in a progressive clubfoot position (also referred to as the reverse Ponseti method). Closed or minimally open reduction with Kirschner wire fixation of the talonavicular joint and percutaneous tendon Achilles lengthening is then performed [2,13].

As stated, the first step to treat CVT is manipulation, which involves gently stretching the foot in plantarflexion, adduction, and inversion while applying counterpressure with the opposite thumb to gently shift the talus dorsally and laterally. This effectively assists in opening the talonavicular joint and facilitates reduction. It also prevents compression of the dorsally displaced navicular into the talus, which would promote hypoplastic development. Avoiding calcaneal manipulation is essential to allow the calcaneus to correct from a valgus to a varus position [1].

After manual manipulation, the foot is maintained in the desired position with a long-leg plaster cast. The cast is carefully molded around the talar head, malleoli, and above the calcaneus posteriorly with the knee set at approximately 90 degrees of flexion. Casting is repeated every one to two weeks in the outpatient setting but ideally should be initiated within the first few months of life. On average, about five casting sessions are required to successfully reduce the talonavicular joint, with the foot being placed further into equinovarus each session [1]. Extreme equinovarus in the final cast should allow for acceptable stretching of the soft tissues dorsolaterally.

Once reduction is achieved through serial casting, the patient undergoes surgical stabilization of the talonavicular joint. This is achieved using K-wires followed by percutaneous Achilles tendon release. If the talonavicular joint remains displaced, a small capsulotomy can be made anterior to the subtalar to allow the placement of an instrument, such as an elevator, to complete the reduction [1]. With the talonavicular joint successfully reduced, another K-wire is fired retrograde across the joint to maintain stability. Additional tenotomies or tendon lengthenings can be performed for any residual contractures after serial casting. The structures most affected by residual contracture are the peroneus brevis, tibialis anterior, and dorsal extensor tendons [1]. Finally, an Achilles tenotomy is performed to address any lingering hindfoot equinus. 

Following surgical correction, a long leg cast is applied with the ankle and forefoot placed in neutral. The cast is removed at two weeks postoperatively to manipulate the ankle to 10 degrees of dorsiflexion before being placed back into a cast. The K-wire is removed at four to six weeks postoperatively, and the patient transitions to a shoe and bar brace system. General recommendations are to wear the brace full-time for three to four months, then transition to wearing it only at night for the next few years to prevent relapse. Various static and dynamic bars are available to facilitate an active range of motion of the knees and ankles. 

It is evident that treatment success relies heavily on patient and parent compliance. Educating parents on how to perform foot-stretching exercises several times a day that emphasizes ankle plantarflexion and foot adduction is crucial to maintaining foot flexibility. Solid ankle-foot orthoses are additionally used for daytime support when the patient is walking. Bracing off of a bar is generally not indicated for patients with an isolated vertical talus [1].

The evolution of treatment for congenital vertical talus

Historically, nonoperative treatment consisted of serial manipulation and casting [17]. Over the years, studies concluded that serial casting alone was not sufficient, and that combination with surgical treatment resulted in superior outcomes with reduced relapse [8]. 

Surgical treatment of CVT was first described in 1939 by Lamy and Weissman, who recommended excision of the talus for definitive treatment [14,20]. This was followed by Eyre-Brooke in 1967 who advocated the excision of the navicular definitive treatment [14]. Suffice it to say, that neither option is now an accepted form of treatment. Surgical treatment of CVT was then performed using either a single-stage or two-stage extensive soft-tissue release [1,21-23]. According to Coleman et al., the two-stage approach consisted of first lengthening the contracted dorsolateral tendons, releasing the dorsolateral capsular contractures, and reducing the talonavicular and subtalar joint complexes. The second stage consisted of tendon Achilles lengthening as well as lengthening of the peroneal tendons and posterolateral capsular release [4].

The single-stage approach to CVT correction was a combination of the two aforementioned stages into a single operation, either through a medial [24] or dorsal [22] approach. In 1997, Stricker and Rosen published their results of 20 feet (13 patients) with CVT treated using a single-stage dorsal approach through an oblique incision centered over the anterior inferior ankle crease extending from the talonavicular joint laterally to the tip of the fibula [14,21]. Patients in this cohort underwent an extensor hallucis longus and peroneus tertius tenotomy, talonavicular joint reduction and K-wire fixation, and percutaneous Achilles lengthening. The authors evaluated seven clinical and eight radiographic parameters, with an average follow-up of 41 months, and observed good results in 17 feet with fair results in three feet. Importantly they found this technique to be associated with maintained plantigrade correction with a reduction in pain and stiffness [21]. In a landmark study by Mazzocca et al., the authors retrospectively compared radiographic and clinical outcomes in 18 feet (6 patients) treated with a single-stage dorsal approach versus 25 feet (18 patients) treated with two-staged posterior approaches with a minimum three-year follow-up [25]. Outcomes were reported using the Adelaar-Williams-Gould scoring system for CVT [26]. The single-stage dorsal group required no revision operations, had a higher Adelaar clinical score (8.0 vs 6.75), and was associated with shorter tourniquet time (87 vs 123 minutes) compared to the two-staged posterior group. In addition, there were no reported incidences of avascular necrosis in the single-stage dorsal group, compared to 12 (66.7%) patients in the two-stage posterior group. Mazzocca et al. concluded that while both procedures successfully reduced the talonavicular joint, single-stage correction was associated with less surgical time, fewer complications, and improved clinical outcomes [14,25].

Treatment of CVT bears the risk of under-correction, overcorrection, and relapse, regardless of which method has been utilized for correction. The primary issue lies in the accurate reduction of the talonavicular joint, as most under-corrected feet are actually due to a result of incomplete realignment of the navicular and talus. In 2006, Dobbs and colleagues advocated the aforementioned minimally invasive approach which is now the standard of care. The authors highlighted a new manipulation and casting methodology based on the principles by Ponseti in the treatment of clubfoot deformity, now known as the “reverse Ponseti” method, followed by pinning the talonavicular joint with K-wires and percutaneous Achilles tendon release [2,14]. The authors advocated this approach as it avoided extensive surgical releases and therefore minimized the risk of postoperative stiffness. They found that at a minimum two-year follow-up, mean ankle dorsiflexion and plantarflexion were 25 degrees and 33 degrees, respectively, with all postoperative radiographic parameters within normal ranges. The authors did, however, observe recurrent dorsal subluxation of the talonavicular joint in three (27.3%) patients.

Complications

Regardless of the surgical approach, surgical correction of CVT can cause a myriad of complications, including wound necrosis, osteonecrosis of the talus, and under- or overcorrection of the deformity. Long-term complications may also occur, which include stiffness of the tibiotalar and subtalar joints and accelerated degenerative arthritis. These complications are not unique to CVT, and many are frequently reported following soft-tissue releases used to address club foot deformity [1,27].

Arguably the most prevalent complication arises from incomplete correction during initial treatment, resulting in persistent deformity [1,21]. Deformity relapse, while less common, can still occur and is usually managed with repeat casting [1]. Kodros et al. observed higher rates of recurrence in patients with underlying neurologic conditions such as spina bifida [23]. However, in patients over two years old with recurrent deformity, correction with open reduction of the talonavicular joint may be necessary [1]. Even older patients with recurrent deformities may require salvage procedures including subtalar fusion (Grice-Green procedure), triple arthrodesis, and/or talectomy [14,28].

In general, overall outcomes and prognosis following prompt treatment of CVT are favorable, with most patients reporting mild long-term deficits such as foot size asymmetry, calf atrophy, and reduced ankle range of motion. 

## Conclusions

Congenital vertical talus is an uncommon foot deformity that is often missed or misdiagnosed in newborns. This is largely attributed to the challenges in interpreting newborn radiographs and the broad range of possible differential diagnoses associated with rigid foot deformity. However, careful examination of the foot that includes examination for a concurrent hindfoot equinus contracture can improve recognition and diagnosis of CVT, allowing for early intervention and improved treatment outcomes. Historically, surgical management of CVT involved extensive soft tissue releases that were fraught with various complications. Newer techniques focus on serial manipulation and casting followed by minimally invasive fixation and soft tissue releases as needed, which have demonstrated excellent results. To further advance our understanding and management of CVT, it is imperative to explore the relationship between CVT etiology and its correlation with physical exam findings on presentation. This will allow for the development of an enhanced classification system that can more accurately predict patient prognosis and assist clinicians in predicting a patient’s responsiveness to treatment.
